# Poly(rC) binding protein 2 (PCBP2) promotes the viability of human gastric cancer cells by regulating CDK2

**DOI:** 10.1002/2211-5463.12408

**Published:** 2018-03-22

**Authors:** Changyu Chen, Jun Lei, Qiang Zheng, Sheng Tan, Keshuo Ding, Changjun Yu

**Affiliations:** ^1^ Department of General Surgery (Gastrointestinal Surgery) The First Affiliated Hospital of Anhui Medicial University Hefei China; ^2^ Laboratory of Molecular Tumor Pathology School of Life Science University of Science and Technology of China Hefei China; ^3^ Department of Pathology Anhui Medical University Hefei China

**Keywords:** CDK2, gastric cancer, PCBP2, viability

## Abstract

Survival rates for patients with gastric cancer, especially the advanced form, remain poor and the development of targeted treatments is hampered by a lack of efficient biological targets. Poly(rC) binding protein 2 (PCBP2) is an RNA‐binding protein that contributes to mRNA stabilization, translational silencing and enhancement and it has been implicated as a promoter of gastric cancer growth. In the present study, we demonstrated that the expression level of PCBP2 was higher in human gastric cancer tissues compared to adjacent normal gastric tissues. A high level of PCBP2 was correlated with worse postoperative relapse‐free survival and overall survival rates of gastric cancer patients. Small hairpin RNA‐mediated depletion of PCBP2 dramatically decreased the viability of gastric cancer cells. Cyclin‐dependent kinase 2 (CDK2) was positively regulated by PCBP2 via a direct 3′ UTR binding pathway as determined using a ribonucleoprotein immunoprecipitation assay and a biotin pulldown assay. CDK2 mediated the promoting role of PCBP2. These results suggest that PCBP2 acts as an oncogene in human gastric cancer cells and that functionally depleting PCBP2 could be considered as a potential target for gastric cancer therapy.

**Abbreviations**
CDK2: cyclin dependent kinase 2
IP: immunoprecipitation
MTT: 3‐(4,5‐dimethylthiazol‐2‐yl)‐2,5‐diphenyl‐tetrazolium bromide
OS: overall survival
PCBP2: poly(rC) binding protein 2
qRT‐PCR: quantitative RT‐PCR
RFS: relapse‐free survival
shRNA: small hairpin RNA

Gastric cancer, which is the second most common cancer, accounts for almost 10% of all cancer‐related deaths in the world 1, 2, 3. Many advances have been made with respect to diagnosis and treatments in recent years, although the survival rate of patients with gastric cancer, especially advanced gastric cancer, remains poor. The mean postoperative overall survival rate (OS) for advanced gastric cancer is no more than 12 months 4, 5. Surgery, subsequent radiotherapy and chemotherapy are still the main methods for gastric cancer treatment. A lack of efficient biological targets retards the development of targeted therapies 4, 6. Further study of the molecular mechanisms will help provide a better understanding of the initiation and development of gastric cancer, as well as enable the development of new clinical diagnostic and therapeutic methods.

Poly(rC) binding protein 2 (PCBP2), a 39 kDa protein, is a member of the α‐globin mRNP stability complex 7, 8. As a generally known RNA‐binding protein, PCBP2 possesses three K homology domains, which are the main RNA‐recognition regions 9, 10. As a result of the RNA‐binding characteristic, PCBP2 contributes to mRNA stabilization, translational silencing and enhancement 11, 12. The combined effect of the genes regulated by PCBP2 via a RNA‐binding pathway determines the role of PCBP2 in organisms. As reported previously, PCBP2 binds to many RNA viruses, including poliovirus, poliovirus and HCV, amongst others 13. Recently, PCBP2 was reported to contribute to cell viability and the progression of tumors, such as leukemia and glioma 13, 14. Hu *et al*. 15 demonstrated that PCBP2 promoted gastric cancer growth by suppressing miR‐34a. To the best of our knowledge, the intrinsic mechanism of PCBP2 with respect to promoting human gastric cancer is still unclear and further investigations are necessary.

In the present study, we examined the expression of PCBP2, which was much higher in human gastric cancer tissues compared to adjacent normal gastric tissues, and found that the over‐expression of PCBP2 is correlated with poor survival among gastric cancer patients. Functional depletion of PCBP2 suppressed cell viability and decreased the percentage of cell mitosis in human gastric cancer cells. Moreover, cell cycle‐related protein cyclin‐dependent kinase 2 (CDK2) was found to be up‐regulated by PCBP2. Using a RNP immunoprecipitation (IP) assay and a biotin pulldown assay, PCBP2 was determined to directly bind to the 3′ UTR region of CDK2. CDK2 partly mediated the promoting role of PCBP2 in human gastric cancer cells and the expression levels of PCBP2 and CDK2 were positively correlated in gastric cancer tissues.

## Materials and methods

### Patients and tissue samples

In total, 100 gastric cancer tissue samples and 100 adjacent nontumorous tissue samples were collected from patients with gastric cancer who had undergone surgery in The First Affiliated Hospital of Anhui Medical University (Hefei, Anhui, China) between 2010 and 2012. All of these tissues were diagnosed by senior pathologists in the Department of Pathology of The First Affiliated Hospital of Anhui Medical University. All of these patients were followed up for more than 5 years and their postoperative relapse‐free survival (RFS) and OS were documented. The protocol of clinical tissue‐related study was approved by the Institutional Review Boards of the First Affiliated Hospital of Anhui Medical University and was carried out in accordance with The Code of Ethics of the World Medical Association (Declaration of Helsinki). All patients provided their written informed consent.

### Immunohistochemistry

Immunohistochemistry was carried out to analyze the protein levels of PCBP2 and CDK2 in paraffin sections of human gastric cancer tissues or adjacent normal gastric tissues. As described previously, a two‐step histostaining method (Maixin, Fuzhou, China) and PCBP2 Rabbit Polyclonal antibody (1 : 200; Proteintech Group, Inc., Chicago, IL, USA)/CDK2 Mouse Monoclonal antibody (dilution 1 : 100; Proteintech Group, Inc.) were used in the immunohistochemical study 16, 17. The stained sections were evaluated using an Olympus microscope (Olympus America, Inc., Melville, NY, USA). At least 10% of cells positively stained in the sections were designated as PCBP2‐positive and less than 10% cells positively stained in the sections were designated as PCBP2‐negative. Regarding the semiquantitative analysis of the staining intensity distribution of PCBP2 levels in HGC: 30–60% cells positively stained in the sections were designated as PCBP2‐moderate, less than 30% cells positively stained in the sections were designated as PCBP2‐weak and more than 60% cells positively stained in the sections were designated as PCBP2‐strong. The average staining intensity of gastric cancer tissue samples was 58.1% for PCBP2. For Kaplan–Meier analysis, less than 58.1% cells positively stained in the sections were designated as PCBP2‐low and more than 58.1% cells stained were designated as PCBP2‐high.

### Cell lines and transfection

Human gastric cancer cell lines HGC‐27 and MKN‐45 were obtained from the American Type Culture Collection (Rockville, MD, USA). Both of these two cell lines were maintained in a humidified atmosphere of 5% CO_2_ at 37 °C.

The small hairpin RNAs (shRNAs) used in the present study containing shPCBP2#1, shPCBP2#2 and shControl were synthesized by GenePharma (Shanghai, China). A cell transfection assay was performed as recommended using Lipo2000 (Qiagen, Valancia, CA, USA).

### Western blotting

Protein levels of PCBP2 and CDK2 were detected using western blotting, which was performed essentially as described previously 16, 17. PCBP2 rabbit polyclonal antibody, CDK2 mouse monoclonal antibody (both dilution 1 : 1000; Proteintech Group, Inc.) and GAPDH rabbit polyclonal antibody (dilution 1 : 10 000; Proteintech Group, Inc.) were used. GAPDH was examined as a control.

### Quantitative RT‐PCR (qRT‐PCR)

mRNA levels of CDK2 in HGC‐27 and MKN‐45 with different transfection were detected using qRT‐PCR, which was performed essentially as described previously 16, 17. Primescript RT reagent kit (Takara, Otsu, Japan) and SYBR Premix Ex Taq Kit (Takara) were used 16, 17. GAPDH was examined as a control.

### Cellular viability assays

The cellular viability assays used in the present study, comprising a cell counting assay, 3‐(4,5‐dimethylthiazol‐2‐yl)‐2,5‐diphenyl‐tetrazolium bromide (MTT) assay and cell colony formation assay, were performed essentially as described previously 16, 17. Briefly, for the cell counting assay, cells were planted into six‐well plates 24 h after transfection (10 000 per well) and cells were counted everyday for 5 days. Cell growth curves were drafted in the end. For the MTT assay, cells were planted into 96‐well plates 24 h after transfection (2000 per well) and the MTT evaluation was carried out 72 h later. For the cell colony formation assay, cells were planted into six‐well plates 24 h after transfection (1000 per well) and cell colony formation was evaluated 10–15 days later.

### Flow cytometric analysis

Flow cytometric analysis was performed in HGC‐27 and MKN‐45 cells after transfection to evaluate the cell cycle condition. Cells were harvested 72 h after transfection and the cells were fixed and then incubated in 100 μg·mL^−1^ Rnase A and 50 μg·mL^−1^ propidium iodide for 30 min at room temperature.

### mRNA decay assay

The mRNA decay assay was performed to detect the interaction between PCBP2 protein and CDK2 mRNA. Cells were treated with actinomycin D (10 μg·mL^−1^) 48 h after shPCBP2 transfection and then the cells were harvested at 0, 2, 4, 6 and 8 h after actinomycin D treatment. Total RNA was isolated and the mRNA levels of CDK2 were examined using qRT‐PCR. GAPDH was also detected as a control.

### Luciferase reporter assay

A Dual Luciferase ReporterAssay System (Promega Corp., Madison, WI, USA) was used for the luciferase reporter assay in the present study, which was performed essentially as described previously 17.

### Biotin pulldown assay

A biotin pulldown assay was carried out to detect the binding site of PCBP2 protein in CDK2 mRNA. A MAXIScript T7 kit (Invitrogen, Carlsbad, CA, USA) was used to synthesize the biotinylated transcripts. Streptavidin‐coupled Dynabeads (Invitrogen) were used to isolate the protein–mRNA complex. The proteins in the protein–mRNA complex were examined using western blotting.

### RNP‐IP RT‐PCR

RNP‐IP RT‐PCR was used to examine the binding between PCBP2 protein and CDK2 mRNA, which was performed essentially as described previously 18. Anti‐PCBP2 antibody was used to capture the PCBP2 protein–CDK2 mRNA complex. CDK2 mRNA levels were evaluated using qRT‐PCR. IgG was used as a negative control.

### Statistical analysis

All of the experiments were performed independently at least three times and the reported data represent the mean results. An unpaired two‐tailed *t*‐test was used for the cellular viability assays, flow cytometric analysis, the luciferase reporter assay and qRT‐PCR. Pearson's chi‐squared test was used for the immunohistochemical analysis. Kaplan–Meier curves and a log‐rank test was used for analysis of RFS and OS. *P* < 0.05 was considered statistically significant.

## Results

### The expression of PCBP2 is up‐regulated in gastric cancer and an increased expression of PCBP2 predicts a poor prognosis in patients with gastric cancer

**Figure 1:**
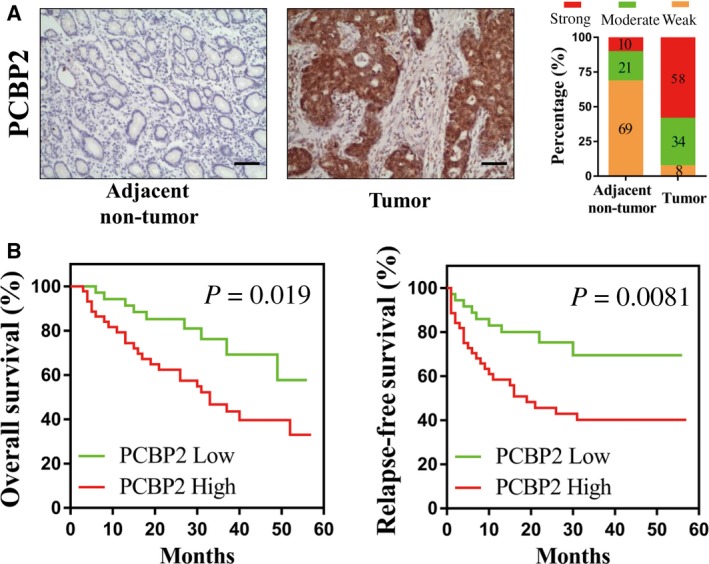
Expression of PCBP2 in tissues from patients and the association of PCBP2 expression with the survival of patients with gastric cancer. (A) Protein levels of PCBP2 in gastric cancer tissues and adjacent normal gastric tissues were detected using immunohistochemistry. Representative photographs are presented. Scale bar = 50 μm. (B) Different RFS and OS between the PCBP2 high group (*n* = 54) and PCBP2 low group (*n* = 46) of those 100 gastric cancer patients who were analyzed using Kaplan–Meier curves.

To study the expression of PCBP2 in human gastric cancer tissues and adjacent normal gastric tissues, 100 gastric cancer tissues and 100 gastric adjacent nontumorous tissues were collected and the protein level of PCBP2 in these tissues were examined using immunohistochemistry. As shown in Fig. 1A, positive signals of PCBP2 protein were predominantly located in the cytoplasm. The expression level of PCBP2 was significantly higher in gastric cancer tissues compared to adjacent nontumorous gastric tissues.

To study the association of PCBP2 expression with RFS and OS rates of patients with gastric cancer, the 100 patients with gastric cancer were followed up for more than 5 years and Kaplan–Meier estimates were conducted. Compared with those patients with lower levels of PCBP2, patients with higher levels of PCBP2 had dramatically poorer RFS and OS rates (RFS rates: *P* = 0.019; OS rates: *P* = 0.002) (Fig. 1B). Therefore, a high level of PCBP2 was associated with a poor prognosis in patients with gastric cancer.

### PCBP2 promoted the viability of human gastric cancer cells

**Figure 2:**
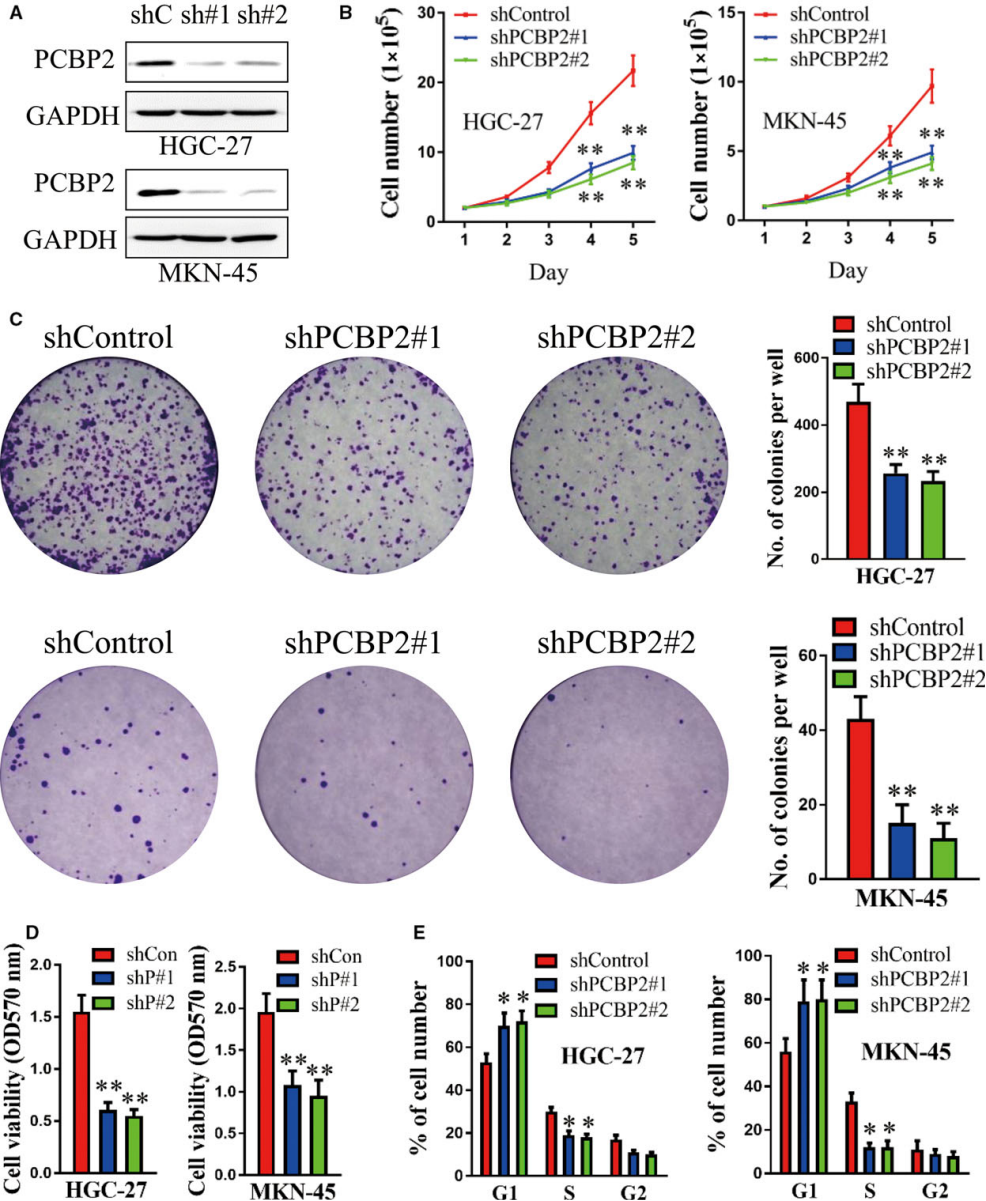
PCBP2 promoted the viability of human gastric cancer cells. (A) Protein levels of PCBP2 were detected after transfection with shPCBP2#1, shPCBP2#2 or shControl in HGC‐27 and MKN‐45 cells using western blotting. GAPDH levels were also detected as a loading control. (B) The cell total number assay was performed in HGC‐27 and MKN‐45 cells after transfection with shPCBP2#1, shPCBP2#2 or shControl. Cells were counted everyday for 5 days and cell growth curves were constructed to evaluate cell growth. (C) Cell colony formation assay and (D) an MTT assay were performed after transfection with shPCBP2#1, shPCBP2#2 or shControl. (E) Flow cytometry was carried out to evaluate the proportions of cells in the G1, S, and G2 phases of the cell cycle in HGC‐27 and MKN‐45 cells after transfection with shPCBP2#1, shPCBP2#2 or shControl. *P < 0.05; **P < 0.01.

Based on the results in clinical tissues, PCBP2 was inferred to be an oncogene in human gastric cancer. To examine the role of PCBP2 in gastric cancer cells, shRNAs (shPCBP2#1 and shPCBP2#2) were stably transfected into HGC‐27 and MKN‐45 cells. As shown in Fig. 2A, the protein level of PCBP2 decreased significantly after transfection with shPCBP2#1 or shPCBP2#2 in both HGC‐27 and MKN‐45 cells compared to shControl. shPCBP2#1 or shPCBP2#2 dramatically decreased total cell number in HGC‐27 and MKN‐45 cells compared to control (Fig. 2B). Concordantly, cell colony formation of both HGC‐27 and MKN‐45 cells significantly deceased after knocking down PCBP2 (Fig. 2C). Moreover, shPCBP2#1 or shPCBP2#2 also dramatically suppressed cell viability in HGC‐27 and MKN‐45 cells, as measured using the MTT assay (Fig. 2D). In addition, as determined by flow cytometry, shPCBP2#1 or shPCBP2#2 significantly increased the percentage of cells in the G1 phase and decreased the percentage of cells in the S phase both in HGC‐27 and MKN‐45 cells (Fig. 2E). As a result, PCBP2 dramatically promoted the viability of human gastric cancer cells.

### CDK2 expression was regulated by PCBP2

**Figure 3:**
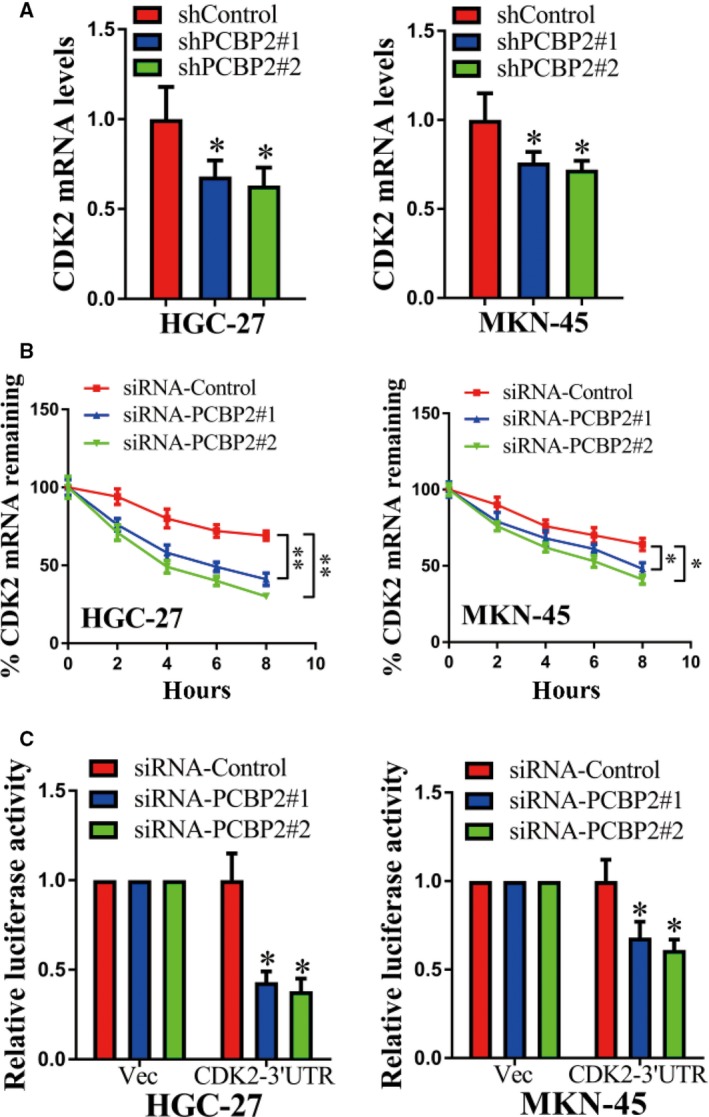
CDK2 expression was positively regulated by PCBP2. (A) mRNA levels of CDK2 were detected after transfection with shPCBP2#1, shPCBP2#2 or shControl in HGC‐27 and MKN‐45 cells using qRT‐PCR. (B) shPCBP2#1, shPCBP2#2 or shControl were transfected into HGC‐27 cells and cells were treated with actinomycin D (10 μg·mL ^−1^) after 48 h. These cells were harvested at 0, 2, 4, 6 and 8 h after actinomycin D treatment. mRNA levels of CDK2 were detected using qRT‐PCR. GAPDH was used as a loading control. (C) Luciferase reporter activities were detected in HGC‐27 cells after co‐transfection with shPCBP2#1/shPCBP2#2/shControl and psi‐CHECK2‐CDK2‐3′UTR/psi‐CHECK2‐Vec, respectively. *P < 0.05; **P < 0.01.

In a further study, several candidate genes were selected for examination after transfection with shPCBP2#1 and shPCBP2#2 in HGC‐27 and MKN‐45 cells. As shown in Fig. 3A, the mRNA levels of CDK2 decreased significantly after transfection with shPCBP2#1 or shPCBP2#2 compared to control. Moreover, the rate of CDK2 mRNA decay dramatically increased after depletion of PCBP2 (Fig. 3B). In addition, the depletion of PCBP2 significantly decreased the level of CDK2 3′ UTR luciferase reporter activity (Fig. 3C). As reported previously, CDK2 participated in cell cycle‐related behaviors and contributed to cell viability 19, 20. Therefore, CDK2 expression was positively regulated by PCBP2 and PCBP2 increased CDK2 transcript stability in a post‐transcriptional manner. CDK2 might mediate the tumor‐promoting role of PCBP2 in human gastric cancer cells.

### CDK2 was regulated by PCBP2 via 3′ UTR binding

**Figure 4:**
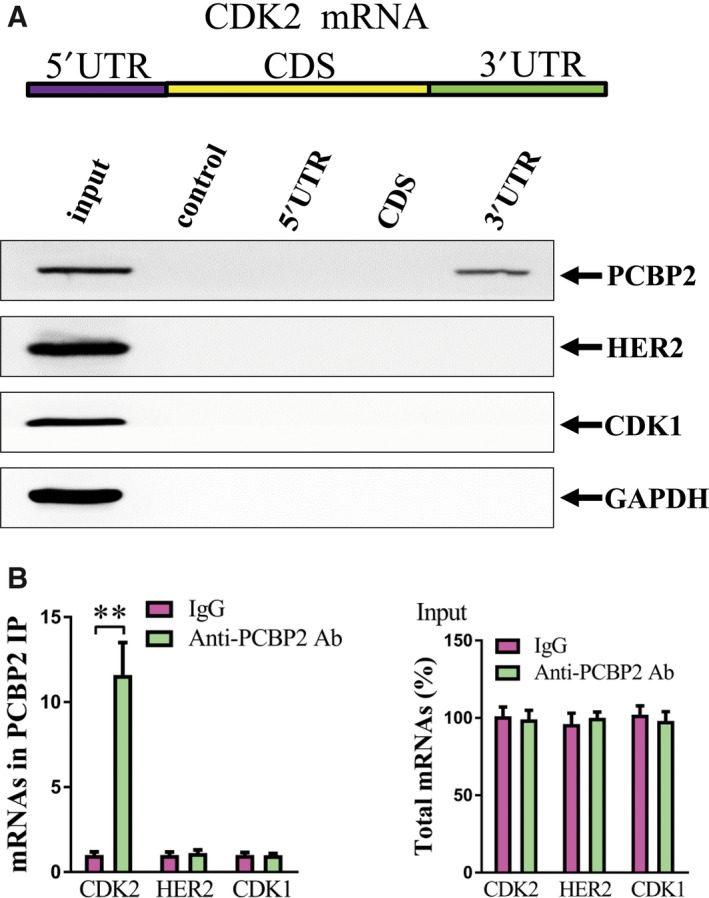
PCBP2 directly bound to CDK2 3′ UTR. (A) Biotinylated RNA pulldown assay. The biotinylated different regions of CDK2 mRNA were incubated with HGC‐27 cell lysates and the mRNA regions interacting with PCBP2 were analyzed using western blotting. Both PCBP2 and GAPDH protein levels are presented and GAPDH was used as a control. (B) RNP IP assay. mRNAs of CDK2 and negative control genes (HER2 and CDK1) were captured by anti‐PCBP2 antibody or control IgG and were analyzed using qRT‐PCR. The total input mRNAs of CDK2 and negative control genes (HER2 and CDK1) were also detected. **P < 0.01.

To confirm the molecular mechanism for the regulation of CDK2 by PCBP2, a biotin pulldown assay was performed to detect the specific CDK2 mRNA region to which PCBP2 protein had bound. As shown in Fig. 4A, PCBP2 directly bound to the 3′ UTR region of CDK2, although there were no positive signals in other regions of CDK2 mRNA. Furthermore, a RNP IP assay was carried out using anti‐PCBP2 antibody and the mRNA levels of CDK2 and negative control genes HER2 and CDK1 were detected. Compared with the control IgG precipitated group, anti‐PCBP2 antibody dramatically enriched the CDK2 mRNA. However, there was no significant change of the negative control genes HER2 and CDK1. Furthermore, there was no significant difference of CDK2, HER2 and CDK1 mRNA total level between the anti‐PCBP2 antibody precipitated group and the IgG precipitated group (Fig. 4B). Therefore, CDK2 was regulated by PCBP2 via a direct 3′ UTR binding manner.

### PCBP2 promoted the viability of human gastric cancer cells via CDK2

**Figure 5:**
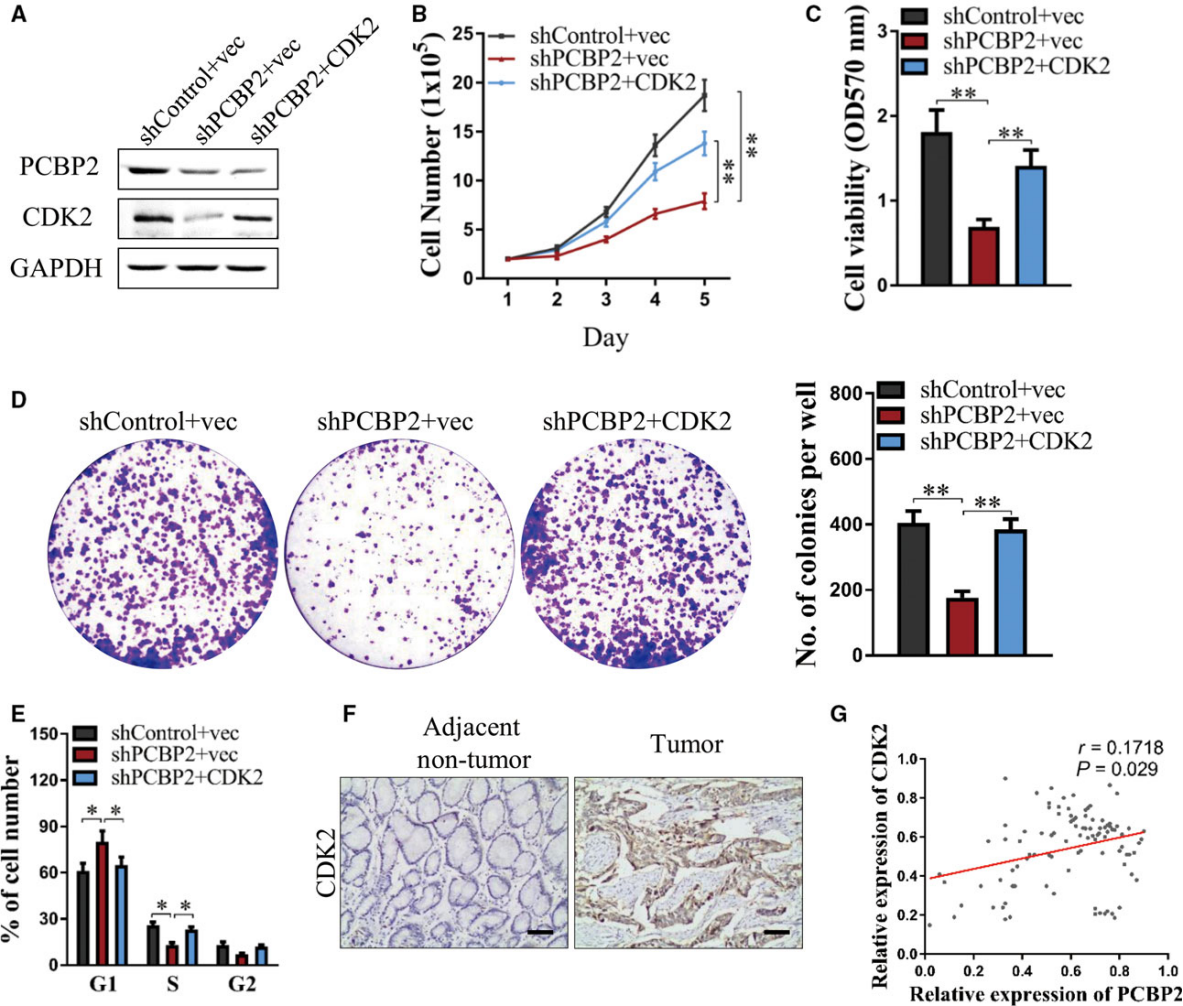
PCBP2 promoted the viability of human gastric cancer cells via CDK2 and expression levels of CDK2 were examined in tissues from gastric cancer patients. HGC‐27 cells were co‐transfected with shPCBP2#1 (designated as shPCBP2) or shControl and CDK2 plasmid or control. (A) Protein levels of PCBP2 and CDK2 were examined using western blotting. GAPDH was used as a loading control. (B) Cell total number assay, (C) MTT assay, (D) cell colony formation assay and (E) flow cytometry (to detect the proportions of cells in the G1, S and G2 phases of the cell cycle) were performed to evaluate cell viability. (F) Protein levels of CDK2 in gastric cancer tissues and adjacent normal gastric tissues were detected using immunohistochemistry. Representative photographs are presented. Scale bar = 50 μm. (G) The correlation between PCBP2 and CDK2 in gastric cancer tissues was analyzed. *P < 0.05; **P < 0.01.

To determine whether PCBP2 promoted the viability of human gastric cancer cells as mediated by CDK2, HGC‐27 cells were co‐transfected with shPCBP2#1 (designated as shPCBP2) or shControl and CDK2 plasmid or control, and cell viability assays were performed. As shown in Fig. 5A, protein levels of PCBP2 decreased significantly after transfection with shPCBP2 + vec or shPCBP2 + CDK2 plasmid compared to control. However, protein levels of CDK2 decreased significantly after transfection with shPCBP2 + vec compared to control and the decrease was abrogated by transfection with pSIN‐CDK2 plasmid. Concordant with the former results, total cell number, cell viability (i.e. as tested by the MTT assay) and cell colony formation all decreased significantly after depletion of PCBP2 in HGC‐27 cells. However, such decreases were abolished by forced expression of CDK2 (Fig. 5B–D). In addition, depletion of PCBP2 dramatically increased the percentage of cells in the G1 phase and decreased the percentage of cells in the S phase in HGC‐27 cells, although these changes were abolished by forced expression of CDK2 (Fig. 5E). Hence, the promoting role of PCBP2 in the viability of human gastric cancer cells was mediated by CDK2.

### Expression of CDK2 in tissues from gastric cancer patients

In a further study, the expression of CDK2 in tissues from gastric cancer patients was examined by immunohistochemistry. In the 100 gastric cancer tissues and 100 adjacent nontumorous tissues, CDK2 exhibited a higher protein level in gastric cancer tissues compared to adjacent nontumorous tissues (Fig. 5F). Moreover, the correlation between PCBP2 levels and CDK2 levels was analyzed in accordance with the percentage of stained cells by immunohistochemistry. As shown in Fig. 5G, PCBP2 and CDK2 were positively correlated in gastric cancer tissues (Pearson's correlation coefficient: *r* = 0.1718, *P* = 0.029).

## Discussion

In the present study, we systematically examined the promoting role of PCBP2 in human gastric cancer cells. In the 200 samples of human gastric tissues (containing 100 cancers and 100 adjacent nontumorous tissues), PCBP2 showed a dramatically higher expression in gastric cancer tissues compared to adjacent nontumorous gastric tissues (67.5% versus 40%, *P* < 0.001). Patients with higher levels of PCBP2 had significantly poorer RFS (*P* = 0.019) and OS (*P* = 0.002) rates compared to patients with lower levels of PCBP2. In gastric cancer cells HGC‐27 and MKN‐45, shRNA‐mediated PCBP2 depletion dramatically decreased cell viability as evaluated using a total cell number assay, cell colony formation assay and flow cytometry. Concordantly, Hu *et al*. 15 reported that PCBP2 promoted cell growth of gastric cancer. PCBP2 knockdown inhibited cell growth both *in vitro* and in an *in vivo* xenograft. Furthermore, it was demonstrated that PCBP2 was correlated with a poor outcome for patients in a tissue study 15. These data all support the results obtained in the present study. In addition, Chang *et al*. 14 demonstrated that PCBP2 suppressed the C/EBP‐driven myeloid differentiation of human myeloid chronic myelogenous leukemia. PCBP2 was also reported to promote glioma cell growth both *in vitro* and in nude mice 21. These results are in accordance with the data of the present study. However, Roychoudhury *et al*. 21 reported that PCBP2 suppressed cell growth and induced cell apoptosis in human oral cancer cells, demonstrating the tissue specificity of PCBP2 in different human tissues.

For the downstream pathway, CDK2 was identified as being positively regulated by PCBP2. The CDK2 performs important roles in cell mitosis. Abnormal over‐expression or activation of CDK2 induces malignant cell viability 19, 20. In the present study, using an mRNA decay assay and a luciferase reporter assay, we determined that PCBP2 interacted with the mRNA of CDK2 and also that depletion of PCBP2 decreased the expression level of CDK2. Moreover, using a RNP IP assay and a biotin pulldown assay, we identified that the region of interaction between PCBP2 protein and CDK2 mRNA is the 3′ UTR region of CDK2. Moreover, we determined that CDK2 mediated the promoting role of PCBP2 in human gastric cancer cells. As reported previously, CDK2 is involved in cell cycle regulation by miR‐302b and promotes the progression of gastric cancer 19. Liang *et al*. 22 demonstrated an over‐expression of cyclin E and that CDK‐2 promoted tumor initiation, progression and the metastasis potential of human gastric cancer 22. Iseki *et al*. 23 reported that adeblock of CDK2 by cyclin‐dependent kinase inhibitors suppressed the viability of human gastric cancer cells. All of these results support the findings of the present study. Moreover, CDK2 was reported to promote cell viability and tumor progression in many other human cancers, including breast cancer 24, 25, colorectal cancer 26, squamous cell carcinoma of the head and neck 27, neuroblastoma 28 and nonsmall cell lung cancer 29. These results were also concordant with those of the present study. In addition, we have shown that the protein levels of CDK2 were dramatically higher in gastric cancer tissues compared to adjacent nontumorous tissues. PCBP2 and CDK2 were positively correlated in gastric cancer tissues. Therefore, PCBP2 facilitated the viability of gastric cancer cells specifically by regulating CDK2. The PCBP2–CDK2 pathway was found to play an important role in human gastric cancer.

In summary, the present study examined the oncogenic role of PCBP2 in human gastric cancer cells. Systematical *in vitro* cell functional experiments and tissue studies from gastric cancer patients were performed. A high expression level of PCBP2 was associated with a more malignant nature of gastric cancer cells and a worse outcome for patients with gastric cancer. CDK2 was the downstream gene regulated by PCBP2 and the regulating mode was via direct 3′ UTR binding. PCBP2 associated with CDK2 could be used as a potential biomarker for gastric cancer therapy.

## Author contributions

CC, JL and ST conceived and designed the experiments. CC and JL performed the experiments. QZ and ST analyzed the data. KD and CY wrote and revised the manuscript. All authors read and approved the final manuscript submitted for publication.
